# Bioinformatic characterization of STING expression in hematological malignancies reveals association with prognosis and anti-tumor immunity

**DOI:** 10.3389/fimmu.2025.1477100

**Published:** 2025-02-05

**Authors:** Xiang-mei Wen, Zi-jun Xu, Ji-chun Ma, Min-jie Zhang, Ye Jin, Jiang Lin, Jun Qian, Yuan-yuan Fang, Shu-yu Luo, Zhen-wei Mao

**Affiliations:** ^1^ Laboratory Center, Affiliated People’s Hospital of Jiangsu University, Zhejiang, Jiangsu, China; ^2^ Zhenjiang Clinical Research Center of Hematology, Affiliated People’s Hospital of Jiangsu University, Zhenjiang, Jiangsu, China; ^3^ The Key Lab of Precision Diagnosis and Treatment in Hematologic Malignancies of Zhenjiang City, Affiliated People’s Hospital of Jiangsu University, Zhenjiang, Jiangsu, China; ^4^ Department of Hematology, Affiliated People’s Hospital of Jiangsu University, Zhenjiang, Jiangsu, China

**Keywords:** STING, hematological malignancies, prognosis, tumor immune microenvironment, immunotherapy

## Abstract

**Introduction:**

Stimulator of interferon response cGAMP interactor (STING) is essential for both innate and adaptive immunity. However, a comprehensive molecular characterization of STING expression across hematological malignancies is lacking.

**Methods:**

In this study, the pan-blood-cancer landscape related to STING expression was identified using the GTEx, CCLE, Hemap, and TCGA databases, and the potential value for predicting prognosis was investigated. The relationship between STING expression and immune cell enrichment was assessed in the Hemap database. Moreover, the value of STING in predicting the efficacy of immunotherapy was validated using tumor immune dysfunction and exclusion (TIDE) biomarkers and real-world immunotherapy datasets.

**Results and Discussion:**

STING was found to be relatively highly expressed in acute myeloid leukemia (AML) and chronic myeloid leukemia, with higher STING expression correlated with poorer prognosis in AML. STING expression was positively correlated with immune-related pathways such as IFN-gamma response, IFN-alpha response, and inflammatory response. Cytolytic score and STING expression were positively correlated in some hematological tumors, especially chronic lymphocytic leukemia and mantle cell lymphoma. Interestingly, STING expression was negatively correlated with TIDE biomarkers in AML, suggesting that AML patients with a high STING expression level may benefit from immunologic treatment. Our findings contribute a molecular characterization of STING across hematological malignancies, facilitating the development of individualized prognosis and treatment strategies.

## 1 Introduction

Stimulator of interferon genes (STING), encoded by the *STING* gene, is a 378 amino-acid protein that contains three functional domains, namely, four N-terminal transmembrane helices, a central globular domain, and a C-terminal domain (1, 2). STING, also known as TMEM173, MITA, and MPYS, is a type I IFN stimulator that acts as an endoplasmic reticulum adaptor protein, playing an important role in innate immune signaling (3, 4). The innate immune system serves as the first line of host defense that can sense and respond to multiple danger signals from external pathogens or internal neoplasms, resulting in the secretion of inflammatory cytokines and the maturation and activation of proximal antigen-presenting cells (5, 6).

Cyclic GMP-AMP synthase (cGAS) is a direct cytoplasmic DNA sensor that can generate the second messenger cyclic guanosine monophosphate-adenosine monophosphate (cGAMP) and recruit STING to initiate a series of downstream reactions (7–9). Activated STING subsequently recruits and activates tank-binding kinase I (TBK1), which then phosphorylates the transcription factor interferon regulatory factor 3 or nuclear factor kappa B, resulting in its nuclear translocation to promote the transcription of type I IFN genes (9–11). The production of type I IFN further enhances the anti-tumor immune response (12).

STING is crucial for anti-cancer immunity, which involves the activation of immune cells such as dendritic cells, normal-karyotype (NK) cells, and CD8+T cells (13–15). In addition, intra-tumoral STING activation triggers the recruitment of myeloid-derived suppressor cells (MDSCs) and immunosuppression (16). Cancer cells suppress the cGAS/STING pathway during tumor development and progression, leading to tumor immune evasion (10). The cGAS/STING pathway is heterogeneous, with tumor-suppressive or tumor-promoting activity, which offers great potential for the development of antitumor treatments (17, 18). Apoptotic dysfunction in STING-related pathways is found in T-cell-derived tumor cells, and mouse primary T cell leukemia is hyperresponsive to STING agonists, which indicates that STING agonists possess powerful therapeutic potential (19).

Pan-cancer studies have demonstrated that STING is highly expressed in cancer tissues; furthermore, STING expression is closely related to clinical outcomes in some tumor types, suggesting that this protein plays an important role in tumor progression (20, 21). However, the molecular characteristics of STING at the pan-blood-cancer level are lacking. In this study, we aimed to provide novel insights into the functional role of STING in multiple blood cancer types and open up a novel therapeutic strategy. To this end, we comprehensively explored the molecular features, biological effects, and clinical relevance of STING in several hematological malignancies. We also investigated the association of STING with immune cell distribution into the tumor microenvironment (TME) and immune-related genes. Finally, immunotherapy responses were assessed.

## 2 Results

### 2.1 “Landscape” of transcriptional alterations of STING across hematological malignancies

**Figure 1:**
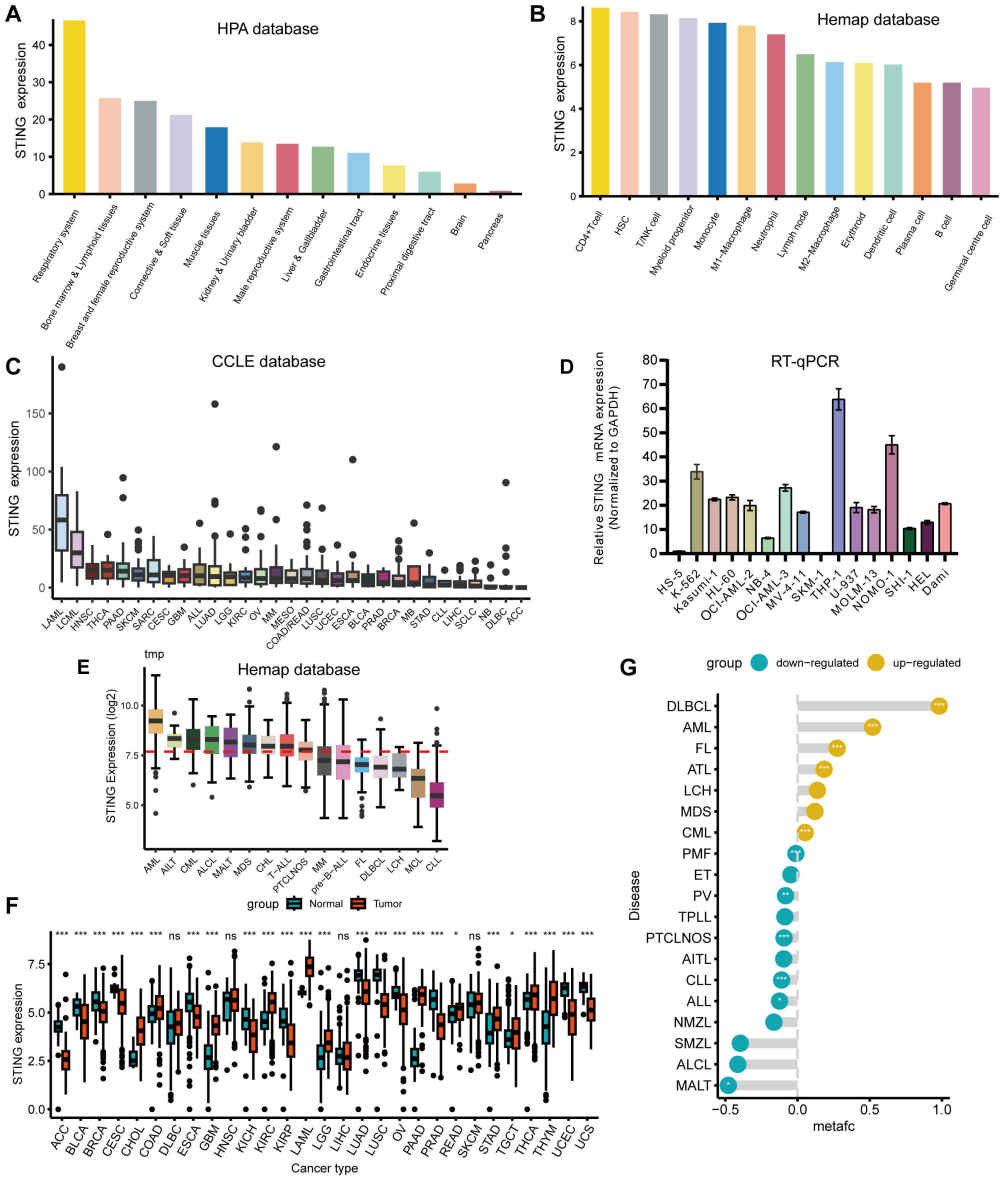
Variation in expression of STING. **(A)** Utilizing the HPA database, STING exhibited high expression levels in respiratory system tissues, bone marrow, and lymphoid tissues. **(B)** STING expression was most pronounced in CD4+ T cells, followed by HSCs and T/NK cells in the Hemap database. **(C)** Analysis of the CCLE database indicated that STING expression levels were comparatively elevated in AML and CML compared with other tumor cell lines. **(D)** RT-qPCR analysis demonstrated a markedly increased expression of STING in the THP-1, NOMO-1, and K562 cell lines from our laboratory. **(E)** STING was overexpressed in AML compared with other hematological malignancies when exploring STING expression in the Hemap database. **(F)** The expression status of the STING gene in various types of cancers in TCGA and GTEx databases. **(G)** Differential expression of STING between hematological malignancies and normal tissues using the Pan-Hem-Diff data. (*P < 0.05, **P < 0.01, ***P < 0.001).

We first assessed the expression of STING in normal tissues using the Human Protein Atlas (HPA) database, and found that STING was expressed at high levels in tissues of the respiratory system, bone marrow and lymphoid tissues, as well as tissues of the breast and female reproductive system (**Figure 1A**). We also explored the onset of STING expression in the blood cells, and STING was intensely expressed in CD4+ T cells followed by hematopoietic stem cells (HSCs) and T/NK cells according to the Hemap database (**Figure 1B**). CCLE database analysis showed that STING was relatively highly expressed in acute myeloid leukemia (AML) and chronic myelogenous leukemia (CML) relative to most cancer cell lines (**Figure 1C**). Subsequently, we collected 16 leukemia cell lines in our laboratory, extracted RNA, and assessed the expression levels of STING using Real-time quantitative PCR (RT-qPCR). Our analysis revealed a significantly elevated expression of STING in the THP-1, NOMO-1, and K562 cell lines (**Figure 1D**). Next, Hemap database analysis confirmed that STING was clearly overexpressed in AML compared with that in other hematological malignancies (**Figure 1E**). Moreover, pan-cancer analysis based on the TCGA and GTEx databases showed that *STING* mRNA was significantly differentially expressed in both normal and tumor tissues in 25 out of 29 tumor types, including AML (**Figure 1F**). Furthermore, STING was differentially expressed across hematological malignancies, with diffuse large B-cell lymphoma (DLBCL) and AML showing the most prominent upregulation (**Figure 1G**). In AML, patients with high STING expression harbored more *FLT3 and DNMT3A* mutations (*P* = 0.038 and *P* = 0.047, respectively) but fewer *RUNX1* and *TP53* mutations (*P* = 0.048 and *P* = 0.018, respectively) than those with low STING expression (**Supplementary Figure S1**).

### 2.2 Prognostic value of STING in hematological malignancies

**Figure 2:**
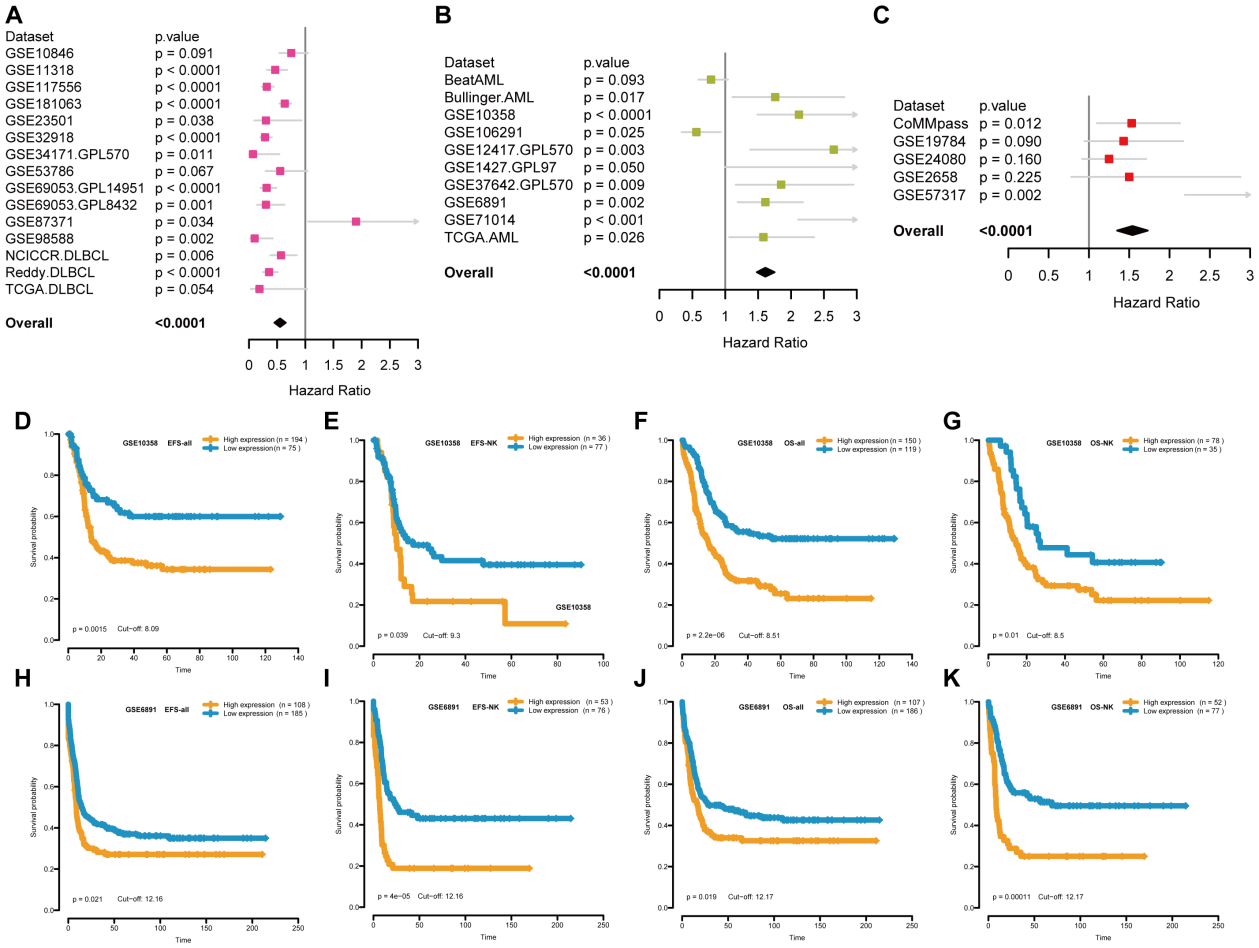
The relationship between STING expression and patient prognosis. **(A-C)** Forest plots of the relationship between STING expression and survival prognosis in DLBCL patients **(A)**, AML patients **(B)** and MM patients **(C)** through a meta-analysis of Cox regression values, where high-risk genes have HR>1 and low-risk genes have HR<1. **(D-G)** Kaplan-Meier curves for EFS and OS of AML patients in GSE10358. **(H-K)** Kaplan-Meier curves for EFS and OS of AML patients in GSE6891. EFS = event-free survival; OS = overall survival; NK = normal karyotype. Orange and blue indicate high and low STING expression groups, respectively.

This study investigated the relationship between STING expression and prognosis of patients with hematological malignancies. As shown in **Figures 2A–C**, a meta-analysis of Cox regression values (*P* value and hazard ratio (HR)) showed that *STING* is a low-risk gene for DLBCL (**Figure 2A**) with HR < 1 in GSE11318 (*P* < 0.0001), GSE117556 (*P* < 0.0001), GSE181063 (*P* < 0.0001), GSE23501 (*P* = 0.038), GSE32918 (P < 0.0001), GSE34171 GPL570 (*P* = 0.011), GSE69053 GPL14951 (*P* < 0.0001), GSE69053 GPL8432 (*P* = 0.001), GSE87371 (*P* = 0.034), GSE98588 (*P* = 0.002), NCICCR DLBCL (*P* = 0.006), and Reddy DLBCL (*P* < 0.0001). *STING* is a high-risk gene for AML (**Figure 2B**) with HR > 1 in Bullinger AML (*P* = 0.017), GSE10358 (*P* < 0.0001), GSE106291 (*P* = 0.025), GSE12417 GPL570 (*P* = 0.003), GSE1427 GPL97 (*P* = 0.05), GSE37642 GPL570 (*P* = 0.009), GSE6891 (*P* = 0.002), GSE71014 (*P* < 0.001), and TCGA AML (*P* = 0.026). And *STING* is a high-risk gene for multiple myeloma (MM) (**Figure 2C**) with HR > 1 in CoMMpass (*P* =0.012) and GSE57317 (*P* = 0.002). Afterward, we specifically chose the GSE10358 (**Figures 2D–G**) and GSE6891 (**Figures 2H–K**) datasets for Kaplan-Meier analysis due to their relatively large sample sizes, inclusion of both overall survival (OS) and event-free survival (EFS) data, and availability of cytogenetic information. These features allowed us to validate the prognostic value of STING for both OS and EFS, as well as in cytogenetically normal AML subgroups. Kaplan-Meier survival analysis revealed higher STING expression correlated with poorer EFS and OS in both the whole and NK subgroups (**Figures 2D–K**).

### 2.3 Correlation between STING expression and the tumor microenvironment in hematological malignancies

**Figure 3:**
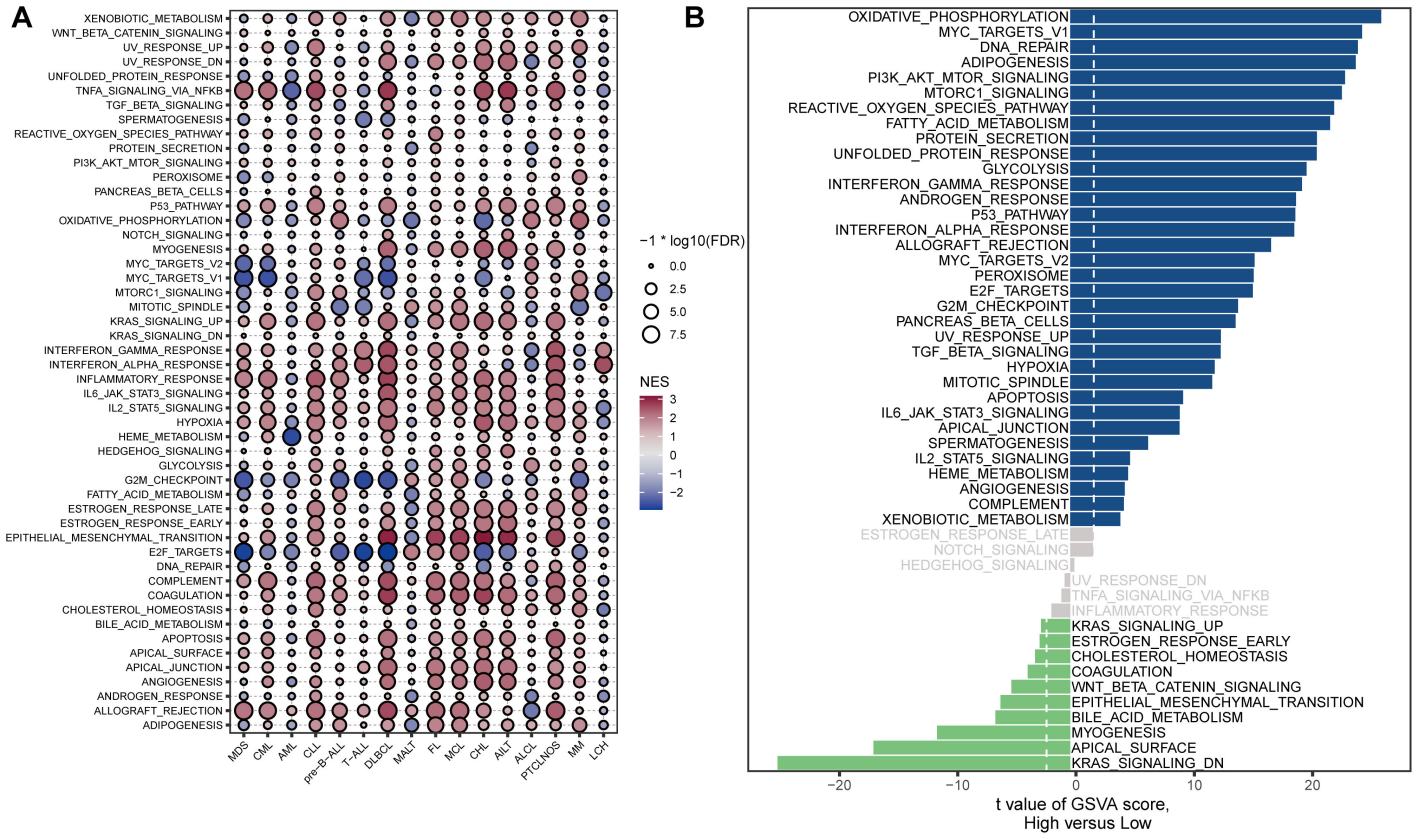
Correlations between STING expression and hallmark signaling pathways. **(A)** Bubble plot of the correlations between STING expression and hallmark gene sets among blood cancer samples in the Hemap dataset. The color indicates the direction of the correlation, positive correlations are shown in red, while negative correlations are represented in blue. The size of the circles indicates the correlation significance level, with larger circles representing lower *P*-values. **(B)** GSVA enrichment analysis using scRNA-seq data from AML patients with low and high STING expression (GSE116256). The signaling pathways on the left were down-regulated, whereas those on the right were up-regulated.

We further investigated the biological characteristics of STING in patients with hematological malignancies using the GSVA package (**Figure 3A**, **Supplementary Table S1**). In general, STING expression was positively correlated with immune-related pathways such as the interferon-gamma (IFN-γ) response, IFN-alpha response, inflammatory response, JAK/STAT3 signaling, complement, and allograft-rejection pathways. In myelodysplastic syndrome (MDS), STING expression was positively correlated with TNF-α signaling via NFKB (r = 2.18, *P* < 0.001), allograft rejection (r = 2.13, *P* < 0.001), inflammatory response (r = 1.96, *P* < 0.001), interferon alpha response (r = 1.80, *P* < 0.001), and interferon-gamma response (r = 1.71, *P* < 0.001). In addition, STING expression was significantly negatively associated with E2F targets (r = −2.86, *P* < 0.001), MYC targets V1 (r = −2.57, *P* < 0.001), G2M checkpoint (r = −2.51, *P* < 0.001), and MYC targets V2 (r = −2.28, *P* < 0.001). In AML, STING expression was positively correlated with allograft rejection (r = 1.35, *P* < 0.05) and negatively associated with HEME metabolism (r = −2.72, *P* < 0.001), and TNF-α signaling via NFKB (r = −2.34, *P* < 0.001). Furthermore, in DLBCL, there was a significant positive correlation between STING expression and epithelial-mesenchymal transition (r = 3.02, *P* < 0.001), TNF-α signaling via NFKB (r = 2.82, *P* < 0.001), coagulation (r = 2.77, *P* < 0.001), inflammatory response (r = 2.70, *P* < 0.001), and interferon-gamma response (r = 2.66, *P* < 0.001). A significant negative correlation was observed between STING and E2F targets (r = −2.93, *P* < 0.001), MYC targets V1 (r = −2.62, *P* < 0.001), G2M checkpoint (r = −2.57, *P* < 0.001), and MYC targets V2 (r = -2.16, *P* < 0.001). To further validate the above findings in AML, we performed GSVA enrichment analysis using scRNA-seq data from AML (GSE116256). The bar plot showing the first 50 GSVA results indicated that STING expression was positively correlated with oxidative phosphorylation and MYC targets V1, and negatively correlated with KRAS signaling, apical surface, and myogenesis (**Figure 3B**).

### 2.4 Associations between the TME signatures and STING expression across hematological malignancies

**Figure 4:**
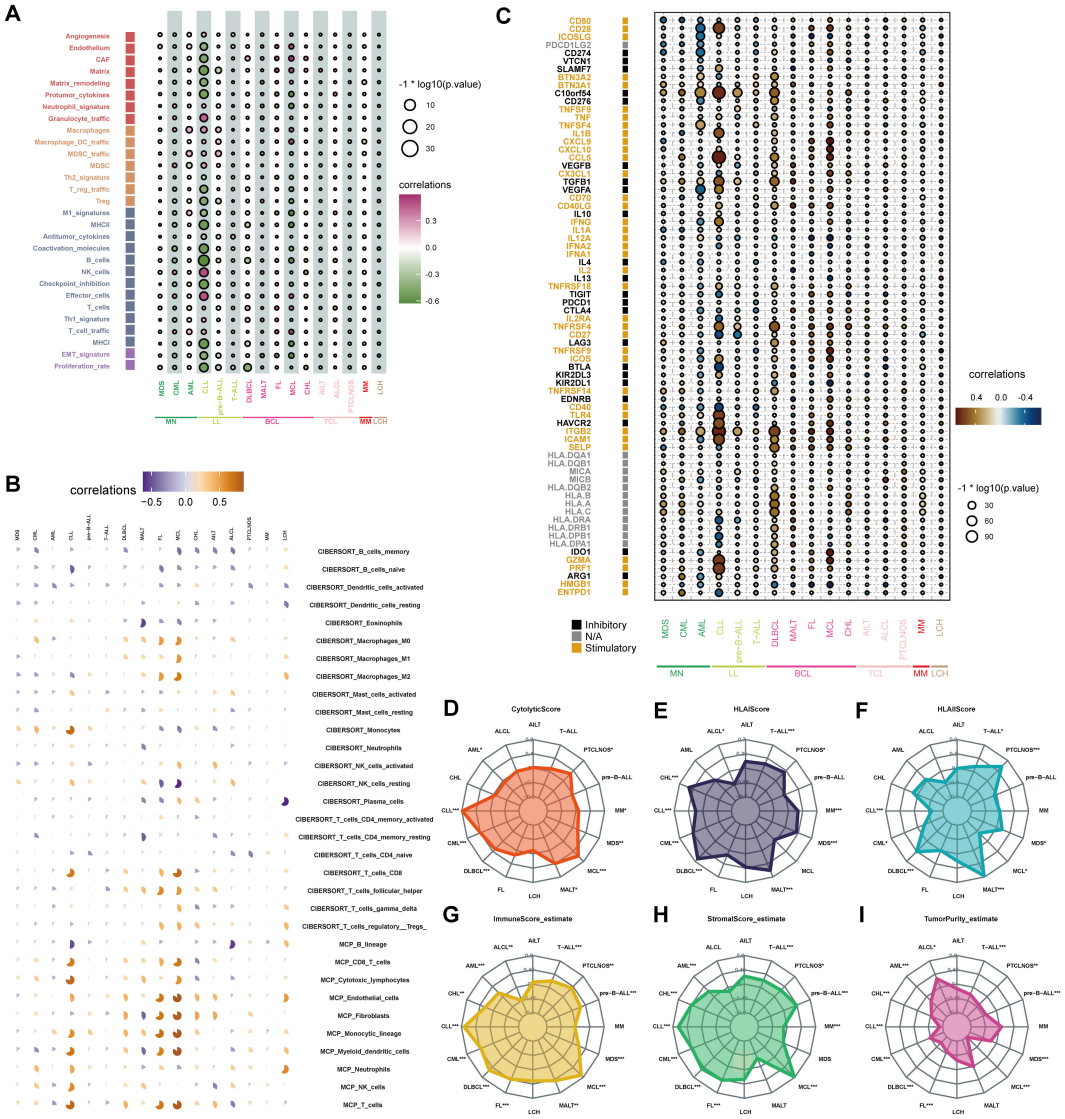
Correlations between the tumor immune microenvironment and STING expression. **(A)** The bubble chart showed the relationship between STING expression and 29 TME signatures across hematological malignancies in the Hemap database. **(B)** Each pie chart showed the correlation between STING expression and immune cell distribution in each hematologic malignancy based on CIBERSORT or MCP-counter analysis. Positive and negative associations are shown on a scale from orange to blue. **(C)** The bubble chart showed the relationship between STING expression and immune genes across hematological malignancies. **(D-I)** Association of cytolytic score **(D)**, HLA I score **(E)**, HLA II score **(F)**, immune score **(G)**, stromal score **(H)**, and tumor purity **(I)** with STING expression in hematological neoplasms. (*p < 0.05, **p < 0.01, ***p < 0.001).

A total of 29 TME-related signatures representing the stromal compartments (e.g., angiogenesis and cancer-associated fibroblasts), pro-tumor microenvironment via macrophages and MDSCs, anti-tumor microenvironment (e.g., B cells and T cells), and cancer cell properties (e.g., EMT signature) were selected by Bagaev et al. (22). The four microenvironment subtypes were conserved across cancer types and could predict response to immunotherapy (22). We explored the association between STING expression and the enrichment of 29 TME-related signatures across hematological malignancies (**Figure 4A**, **Supplementary Table S2**). STING expression was significantly correlated with 29 TME signatures. In chronic lymphocytic leukemia (CLL), STING expression was negatively correlated with stromal-related signatures such as endothelium (r = −0.52, *P* < 0.001), cancer-associated fibroblast (CAF) (r = −0.58, *P* < 0.001), matrix (r = −0.67, *P* < 0.0001), matrix remodeling (r = −0.56, *P* < 0.0001), and protumor cytokines (r = −0.63, *P* < 0.0001). Also, STING expression was positively correlated with macrophages (r = 0.43, *P* < 0.0001), MDSC traffic (r = 0.27, *P* < 0.0001), and MDSC (r = 0.33, *P* < 0.0001). Besides, STING expression was significantly inversely correlated with anti-tumor signatures such as B cells, MHC I, EMT signature, and proliferation rate. Still, it positively correlated with macrophages, in the majority of hematologic malignancies. Thus, correlations between STING expression levels and TME in blood cancers play an important role in tumor growth and survival. Further, CIBERSORT and MCP-counter algorithms were used to analyze the correlation between STING expression levels and the composition of tumor-associated immune cells in diverse blood cancers. Consistent with the 29 TME-related signatures, STING expression was positively correlated with both M1 and M2 macrophages in most hematological malignancies (**Figure 4B**). However, in myeloid cancers such as MDS, CML, and CLL, STING expression was negatively correlated with M2 macrophages (**Figure 4B**), suggesting a potential context-dependent role of STING in shaping the immune microenvironment. These findings underscore the complex interactions between STING expression and macrophage subtypes across different hematological malignancies. Additionally, STING expression was negatively associated with B cell enrichment in the tumor microenvironment (**Figure 4B**). In addition to controlling tumor growth through their effects on the immune microenvironment, B cells can enhance the function of T cells by providing a more conducive environment for their activities (23). The relationship between STING expression and 72 immune genes was also analyzed (**Figure 4C**). STING expression was significantly positively related to that of inhibitory immune genes, including *c10orf54* and *TGFB1*, and negatively related to stimulatory immune genes such as *CD40* and *HMGB1*. In addition, STING expression was positively related to that of stimulatory immune genes such as *ITGB2* and *PRF1* in most hematological tumors. This finding needs further exploration and confirmation.

Afterward, the relationship between STING expression and the immunological microenvironment was investigated. The cytolytic score, which serves as a biomarker of anti-tumor immune activity, is an immune activity score estimated by the average gene expression of *GZMA* and *PRF1* (24). In this study, cytolytic score and STING expression were positively correlated in some hematological tumors, especially CLL and Mantle Cell Lymphoma (MCL) (**Figure 4D**).

HLA class I antigens are crucial for the activation of cytotoxic CD8 +T cells. HLA class II molecules present antigenic peptides to CD4+ T cells, which are critical for antitumor immunity (25). We found that STING expression was positively correlated with HLA I in most blood tumor types, the most prominent being DLBCL, Classical Hodgkin lymphoma (CHL), and mucosa-associated lymphoid tissue (MALT) lymphoma (**Figure 4E**). Furthermore, STING expression was positively correlated with HLA II in MALT, MDS, peripheral T-cell lymphoma-not otherwise specified (PTCLNOS), T-cell acute lymphoblastic leukemia (T-ALL), and DLBCL (**Figure 4F**).

Then, ESTIMATE analysis was performed to determine the association of STING expression with immune scores, stromal scores, and tumor purity. MCL had the highest immune score and stromal score with the lowest tumor purity, while anaplastic large-cell lymphoma (ALCL) had the lowest immune score and stromal score with the highest tumor purity (**Figures 4G–I**).

The above results indicate that STING influences patient prognosis by potentially affecting the activity of immune cells and the immune microenvironment in blood tumors. This observation requires further exploration.

### 2.5 STING expression as a biomarker for immune checkpoint blockade response

**Figure 5:**
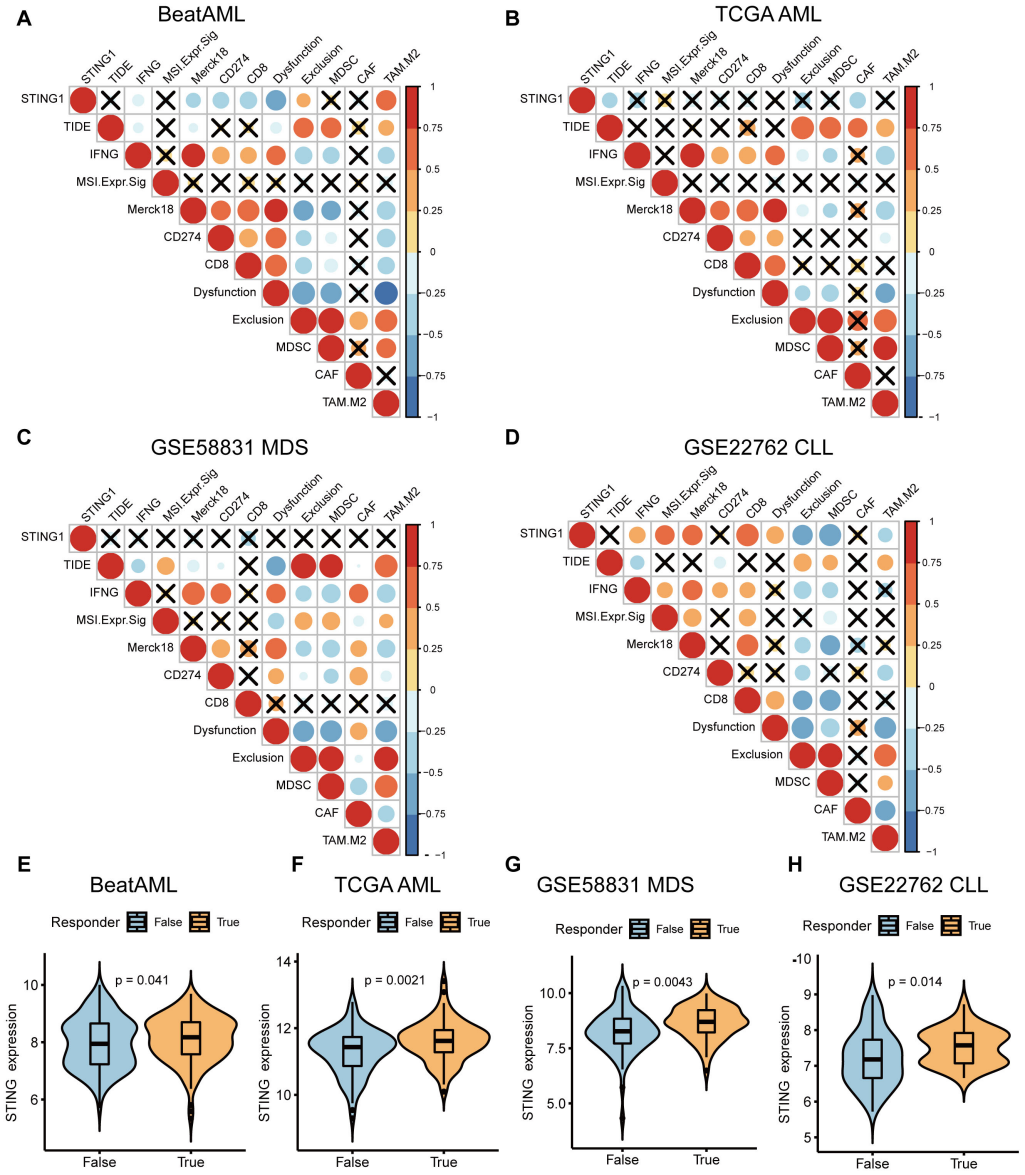
Investigation of the response of STING expression to immunotherapy. **(A-D)** Correlations between STING expression and other immune checkpoints in BeatAML **(A)**, TCGA AML **(B)**, MDS (GSE58831) **(C)**, CLL (GSE22762) **(D)**. **(E-H)** The differences in STING expression profile between the non-responder and responder group in BeatAML **(E)**, TCGA AML **(F)**, MDS (GSE58831) **(G)**, and CLL (GSE22762) **(H)**.

Several studies have reported that patients may benefit from CAR-T therapy, immune checkpoint inhibitors (ICI), and tumor antigen-related vaccines (26–29). Accordingly, we predicted the likelihood of a response to immunotherapy in different STING expression levels using the TIDE algorithm. In the BeatAML database, the results showed a strong negative correlation between STING expression and IFN-γ, Merck18 (T-cell-inflamed signature), CD274, CD8, and T-cell dysfunction-score signatures, whereas a strong positive correlation was observed between STING expression and T-cell exclusion signatures, including exclusion, and the M2 subtype of tumor-associated macrophages (TAMs) (**Figure 5A**). In addition, according to the TCGA AML database, STING expression was negatively correlated with biomarkers of TIDE score and CAF levels (**Figure 5B**). A low TIDE score indicates a lower likelihood of immune evasion, which indicates more benefit from ICI treatment for the patient (30). However, there was no difference in TIDE score, IFN-γ, MSI, Merck18, CD274, CD8, dysfunction, exclusion, MDSC, CAF, and M2 between STING low-expression and STING high-expression groups in the GSE58831 MDS dataset (**Figure 5C**). Furthermore, different results were found in GSE22762 CLL, and STING expression was significantly positively related to IFN-γ, MSI, Merck18, CD8, and T-cell dysfunction and significantly negatively related to T-cell exclusion, MDSC, and TAM M2 (**Figure 5D**).

We further examined the relevance of STING expression in relation to therapeutic response to potential ICB. Excitingly, significant differences in STING expression were observed between the responder and non-responder groups across AML, MDS, and CLL (**Figures 5E–H**). These findings indirectly indicate that STING expression plays a critical role in mediating the immunotherapy response, and patients with high STING expression respond better to immunotherapy.

**Figure 6:**
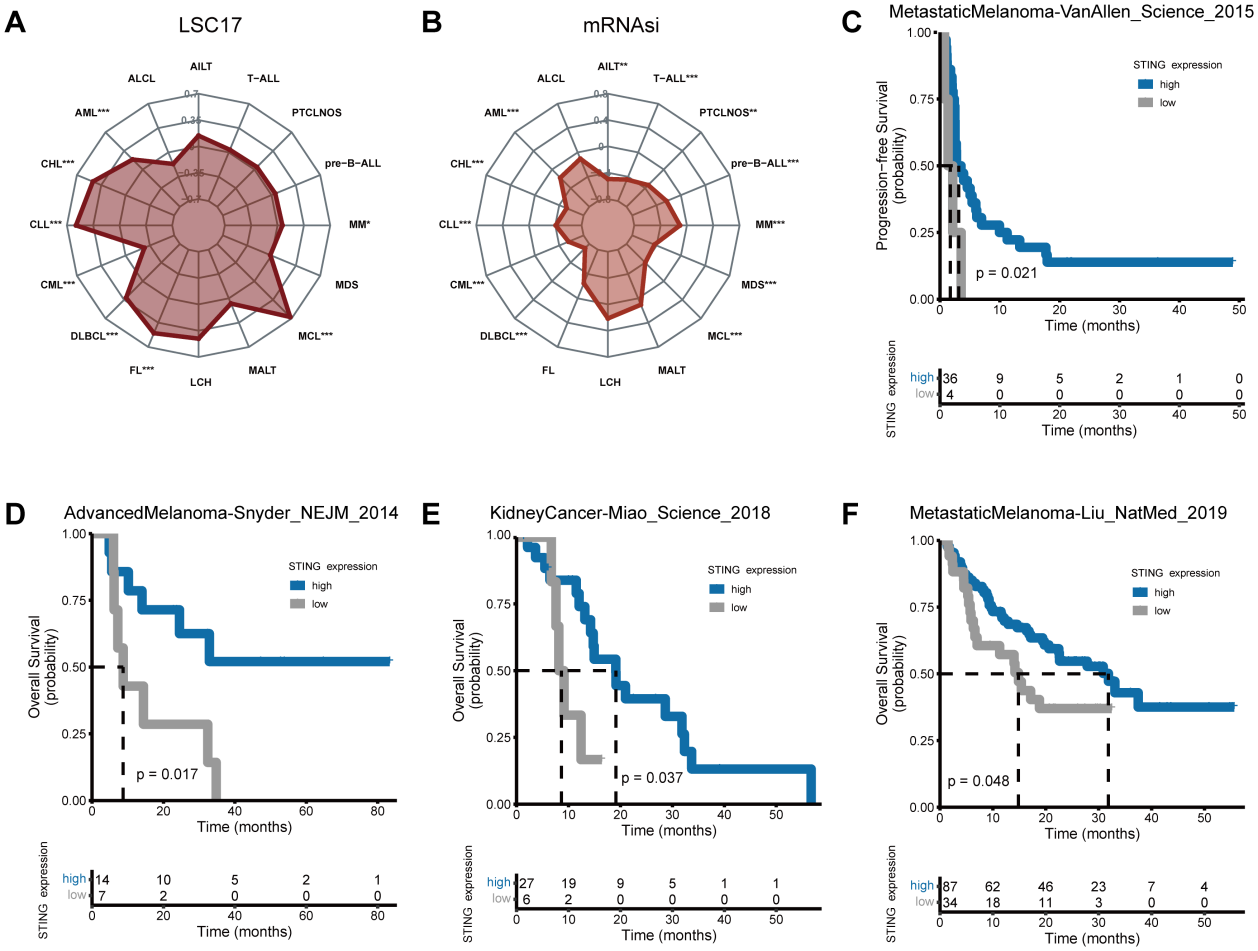
Stemness and response of STING expression to immunotherapy. Correlations of **(A, B)** LSC17 **(A)** and mRNAsi **(B)** score with STING expression in hematological neoplasms. **(C-F)** Patients with higher STING expression exhibited stronger responses to ICB in real-world immunotherapy cohorts encompassing four cancer types. (*p < 0.05, **p < 0.01, ***p < 0.001).

The LSC17 score had strong prognostic value and contributed to the accurate prediction of initial therapy resistance (31). STING expression was positively related to the LSC17 score in CLL, DLBCL, follicular lymphoma (FL), MCL, MM, and AML, but negatively related to the LSC17 score in CML (**Figure 6A**). Patients with higher LSC17 scores had stronger myelodysplasia, and higher recurrence rates, and experienced lower chemotherapeutic effectiveness (31).

To depict the stemness of STING expression in hematological tumors, the mRNA expression-based stemness index (mRNAsi) was evaluated. Significant negative correlations between mRNAsi and STING gene expression levels were found in AML, CHL, CLL, CML, DLBCL, MCL, MDS, MM, pre-B cell acute lymphoblastic leukemias (pre-B-ALL), PTCLNOS, T-cell acute lymphocytic leukemia (T-ALL), and angioimmunoblastic T-cell lymphoma (AITL) (**Figure 6B**).

Using four independent ICB cohorts from four cancer types (**Figures 6C–F**), Kaplan-Meier survival analysis was performed. The MetastaticMelanoma-Liu_NatMed_2019 cohort included anti-PD1-treated metastatic melanoma patients; the AdvancedMelanoma-Snyder_NEJM_2014 cohort included advanced melanoma patients treated with anti-CTLA-4 therapy; the KidneyCancer-Miao_Science_2018 cohort consisted of kidney cancer patients treated with PD-1 or PD-L1 blockade therapy alone or in combination with anti-CTLA-4 therapy; and the MetastaticMelanoma-VanAllen_Science_2015 cohort included anti-CTLA-4-treated metastatic melanoma patients. Results showed that low STING expression was correlated with poor progression-free survival (PFS) and OS across these cohorts, suggesting that STING expression may serve as a biomarker of ICB response in solid tumors.

## 3 Discussion

The role of the cGAS-STING pathway in tumors is still controversial. Activation of the cGAS-STING signaling pathway is bidirectional, causing immune-supporting cells to play antitumor roles, while also producing an immunosuppressive environment that promotes tumor growth and metastasis (32). On the one hand, the cGAS-STING pathway plays a vital role in antitumor immunity and may be an attractive anti-cancer immunotherapeutic drug target (33). On the other hand, increasing evidence suggests that the cGAS-STING pathway induces an immunosuppressive tumor microenvironment (34, 35). However, the role of STING in hematologic malignancies is not well understood. Hence, we attempted to elucidate the expression patterns, clinical characteristics, prognostic value, and relationship with immune scores and immune cell compositions of *STING* expression levels as well as its correlation with immunotherapy response across hematologic malignancies.

In this study, STING exhibited a hematologic-tissue-specific expression pattern across normal and malignant tissue/cell types. STING was largely dysregulated in blood cancers, with upregulation in myeloid malignancies such as AML and CML. STING has also been found to be upregulated in AML patients compared with that in healthy controls (36). It has been shown that STING is more highly expressed in PTCL than in normal lymph nodes (37). We also investigated the effect of the abnormal expression of STING on prognosis in three hematologic malignancies (DLBCL, AML, and MM). Meta-analyses indicated that higher STING expression positively correlated with patient outcomes in DLBCL, and negatively correlated with patient outcomes in AML and MM. Some scholars have reported that high STING expression is significantly related to lower OS and disease-free survival (DFS) (36). Additionally, AML patients with high STING expression were shown to harbor *FLT3*, *DNMT3A*, and *NPM1* mutations more frequently than patients with low STING expression in this study. Chen et al. reported a significantly higher frequency of *NRAS/KRAS* mutation in patients with high STING expression (38).

We observed that STING was positively correlated with IFN-γ and IL6/JAK/STAT3 signaling in most blood cancers. DNA damage activates the cGAS-STING pathway, which induces the type I IFN response and then triggers IFN-γ generation by NK and T cells (7, 39). Subsequently, the synthesis of cytokines, including CXCL10, is enhanced and the immune response is strengthened (37). Researchers have attempted to induce the expression of type I IFN by upregulating STING to treat AML (38, 40, 41). In esophageal carcinoma, the JAK2/STAT3 signaling pathway can promote cell cycle arrest and mitigate the cytotoxic effects induced by radiation, functioning as a downstream component of the cGAS-STING pathway (42). The TME is a complex environment filled with tumor-infiltrating immune cells (TIICs), tumor cells, blood vessels, fibroblasts, and stromal cells (43). Extensive research has shown that the tumor immune microenvironment (TIME) plays an important role in predicting tumor prognosis, evaluating therapeutic effects, and regulating the development of drug resistance and resistance to apoptosis in tumors (44–47). The activation of STING in the tumor microenvironment leads to IFN-I production, resulting in spontaneous antitumor CD8 + T cell responses (48). A study has shown that STING agonists can lead to cell death in T cells by activating cell stress (49). Therefore, this study assessed whether STING expression in TIME affected the progression of hematological malignancies. We observed that STING expression correlated inversely with anti-tumor signatures such as B cells, MHC I, EMT signature, and proliferation rate in the majority of hematologic malignancies. Besides, cytolytic score and STING expression were positively correlated in CLL and MCL in our study. A low cytolytic score represents weak antitumor immunity related to a poor prognosis in gastric cancer and is also associated with poor outcomes in hepatocellular carcinoma (50, 51).

The LSC17 score was originally derived from AML and has demonstrated strong prognostic value in this malignancy, particularly in predicting therapy resistance and disease recurrence. While the regulation of stemness may vary across hematological tumors due to tumor-specific transcriptional programs, many fundamental features of stemness, including self-renewal, resistance to therapy, and tumor initiation, are conserved across malignancies. For instance, both AML and DLBCL exhibit stem-like populations that contribute to poor prognosis and treatment resistance (31, 52, 53). This conservation of stemness characteristics provides a rationale for extending the LSC17 score, originally validated in AML, to other hematological malignancies such as MDS (54). Similarly, our study shows that stemness-related signatures, such as the LSC17 score, derived from AML, show potential for predicting prognosis and therapeutic response in other hematological malignancies. While the exact molecular pathways driving stemness may differ across cancer types, the underlying biological processes—such as self-renewal, resistance to therapy, and tumor initiation—are often conserved. Thus, gene expression-based stemness scores, like those used in AML, may offer valuable prognostic insights in cancers like CLL, DLBCL, and MCL, where stem-like populations contribute to similar clinical challenges, such as relapse and chemoresistance. Our findings suggest that the LSC17 score correlates with stemness in a range of hematological malignancies, highlighting its potential to serve as a prognostic tool beyond AML.

Immunotherapy is an effective form of therapy in cancer (especially in solid tumors) and targets both innate and adaptive immunity. Several studies have demonstrated the importance of the cGAS-STING pathway in immunotherapy of human malignant tumors (55, 56). We attempted to analyze the association of STING expression with immunotherapy and assess its value in the prediction of response to immunotherapy. STING expression negatively correlated with the TIDE score in AML, indicating more benefit from ICB treatment for the patient. The TIDE score has potential utility in identifying patients who are more likely to benefit from ICB (57). STING expression was higher in responders than in non‐responders across AML, MDS, and CLL. Patients with high STING expression tended to benefit from ICB treatment. This finding may offer valuable insights into the development of effective immunotherapies for patients.

## 4 Materials and methods

### 4.1 Data acquisition

Data on *STING* expression in normal tissues were obtained from the HPA database (https://www.proteinatlas.org/). We also analyzed the expression of *STING* in blood cell types and hematologic cancers by using data from the Hemap dataset (http://hemap.uta.fi/). Levels of STING expression were analyzed across cancer cell lines from the Cancer Cell Line Encyclopedia (https://www.broadinstitute.org/ccle). The combined pan-cancer data for the *STING* gene were sourced from the TCGA, TARGET, and GTEx downloaded from the UCSC Xena Browser (https://xenabrowser.net). To confirm whether *STING* expression differs between blood tumors and normal samples, we utilized the Pan-Hem-Diff cohort containing 22 hematologic cancer types with matched tumor and normal samples. Survival information for patients with AML, DLBCL, and MM was downloaded from datasets containing survival information, including the GEO database (https://www.ncbi.nlm.nih.gov/geo/), cBioPortal for Cancer Genomics (http://www.cbioportal.org/), GDC data portal (https://portal.gdc.cancer.gov/), and PREdiction of Clinical Outcomes from Genomic Profiles (PRECOG, https://precog.stanford.edu/). The *STING* gene mutation data was also obtained from the cBioPortal for Cancer Genomics (http://www.cbioportal.org/). Our analyses covered major subtypes of myeloid leukemias, myelomas, B and T cell lymphomas, and lymphoid leukemias, including AML, AITL, ALCL, T-ALL, CML, CLL, CHL, DLBCL, FL, LCH, MM, MDS, MCL, MALT, PTCLNOS, and pre-B-ALL.

### 4.2 Real-time quantitative PCR

Total RNA was extracted from cell lines by using Trizol reagent (Invitrogen, USA). Reverse transcription of RNA was performed to generate cDNA with a Reverse Transcription Kit (Takara, Japan). RT-qPCR was conducted to determine the expression levels of STING in the 16 leukemia cell lines from our laboratory using SYBR Green Master Mix (Takara, Japan). The upstream and downstream primer sequences were 5’- CACTTGGATGCTTGCCCTC-3’ and 5’- GCCACGTTGAAATTCCCTTTTT-3’ respectively. GAPDH was used as the internal reference gene, and STING transcript levels were calculated using the 2^−ΔΔCT^ method.

### 4.3 Analysis of the correlation between STING expression and clinical prognosis

Patients were divided into STING high and low expression groups based on the best cut-off value of STING expression. The ‘best’ cut-off value for Kaplan-Meier analysis was determined using the maxstat method, implemented in the survminer R package. This approach identifies the cut-off that maximizes the log-rank statistic, providing the most statistically significant division of the cohort into high and low-expression groups. Univariate Cox regression analysis was carried out to examine the prognostic value of STING in DLBCL, AML, and MM. We used the “survcomp” package to conduct meta-analyses and combine *P* values and HR. Genes with HR < 1 predicted better OS, while genes with HR > 1 indicated dismal OS. Kaplan–Meier analysis was utilized to exhibit the correlation of STING expression with OS, PFS, and EFS in patients. Kaplan–Meier survival curves were used to visualize survival outcomes between groups using the “survminer” package. Differential gene expression analyses were conducted and signature score data was obtained using the “limma” R package.

### 4.4 Gene set enrichment analyses

We applied more stringent criteria for dichotomizing the expression of STING across the different blood cancer types to provide more meaningful biological interpretations. Differentially expressed genes (DEGs) were extracted between high STING expression (top 30%) and low STING expression (bottom 30%) groups for each blood cancer type in the Hemap dataset. Then, DEGs were subjected to gene set enrichment analysis (GSEA) using the R package clusterProfiler. Further, we selected hallmark gene sets for functional analysis of GSEA from MSigDB (http://www.broad.mit.edu/gsea/msigdb/). The signature scores in the GSE116256 scRNA-seq dataset were computed using the GSVA package.

### 4.5 Effect of STING expression on the tumor immune microenvironment

According to Bagaev et al., 29 functional gene expression signatures were used to describe pan‐cancer TME characteristics and categorize the patterns into stromal compartments, pro-tumor microenvironment, anti-tumor microenvironment, and cancer cell properties (22). Single-sample gene set enrichment analysis (ssGSEA) was used to calculate the signature scores for the tumor samples in Hemap via the “GSVA” R packages. The CIBERSORT and MCP algorithms were used to quantify the immune cell composition in different blood cancers (58). We used computational algorithms (e.g., CIBERSORT, MCP-counter) to infer immune cell proportions in hematological malignancies based on gene expression data, distinguishing this analysis from immune infiltration typically studied in solid tumors. Immune cell profiling in our study reflects cellular enrichment and relative proportions derived from transcriptomic data rather than physical infiltration into a dense tissue matrix. As part of this analysis, the cytolytic scores and HLA scores were analyzed for the Hemap dataset, as reported previously (59). Then, we calculated the immune scores, stromal scores, and tumor purity for each type of blood cancer using the ESTIMATE algorithm.

### 4.6 Immunotherapy response analysis

ICB response, immune dysfunction, and exclusion status were predicted using the TIDE algorithm (http://tide.dfci.harvard.edu). We also analyzed the correlations between *STING* expression levels and programmed cell death 1/programmed cell death ligand 1 (PD-1/PD‐L1) in AML (BeatAML and TCGA), MDS (GSE58831) and CLL (GSE22762). The LSC17 score was calculated for each blood cancer based on previous studies (60, 61). The stemness index mRNAsi was determined from a previous study (https://bioinformaticsfmrp.github.io/PanCanStem_Web/) (62). To predict patients’ response to ICB therapy in real-world immunotherapy settings, we enrolled four independent immunotherapy cohorts covering three cancer types (metastatic melanoma, advanced melanoma, and kidney cancer) to determine whether there were differences in ICB therapy between high and low STING expression groups.

### 4.7 Statistical analyses

R software was employed for statistical analyses. The correlation between two continuous variables was determined using a non-parametric Spearman test. Categorical data were compared using the chi-square test or Fisher’s exact test. Data visualization was performed using the R packages “ggplot2” and survcomp package (for forest plots). *P* < 0.05 was considered to indicate significance.

## 5 Conclusions

The findings of this study reveal the expression patterns of STING across hematological malignancies, and provide insights into the association of STING expression with prognosis, clinicopathological features, and TME cell enrichment. Our findings suggest that STING might serve as a predictor of clinical outcome and immunotherapy response in patients with hematological malignancies. However, further mechanistic studies are necessary to better understand the functional role of STING in blood cancers.

## Data Availability

The original contributions presented in the study are included in the article/**Supplementary Materials**, further inquiries can be directed to the corresponding author/s.

## Supplementary material

Supplementary Figure 1 Differences in somatic mutations between STING high and low groups in AML. The waterfall plot of somatic mutation features in patients with the high STING expression **(A)** or low STING expression **(B)** in the TCGA AML dataset. The mutation profiles showed the mutation difference of the 24 genes in high and low STING expression groups.
