# Supplementary figures and images for: Bioinformatic characterization of STING expression in hematological malignancies reveals association with prognosis and anti-tumor immunity
Source: Front Immunol. 2025 Feb 5;16:1477100. doi: 10.3389/fimmu.2025.1477100 (PMC11835856; doi:10.3389/fimmu.2025.1477100)

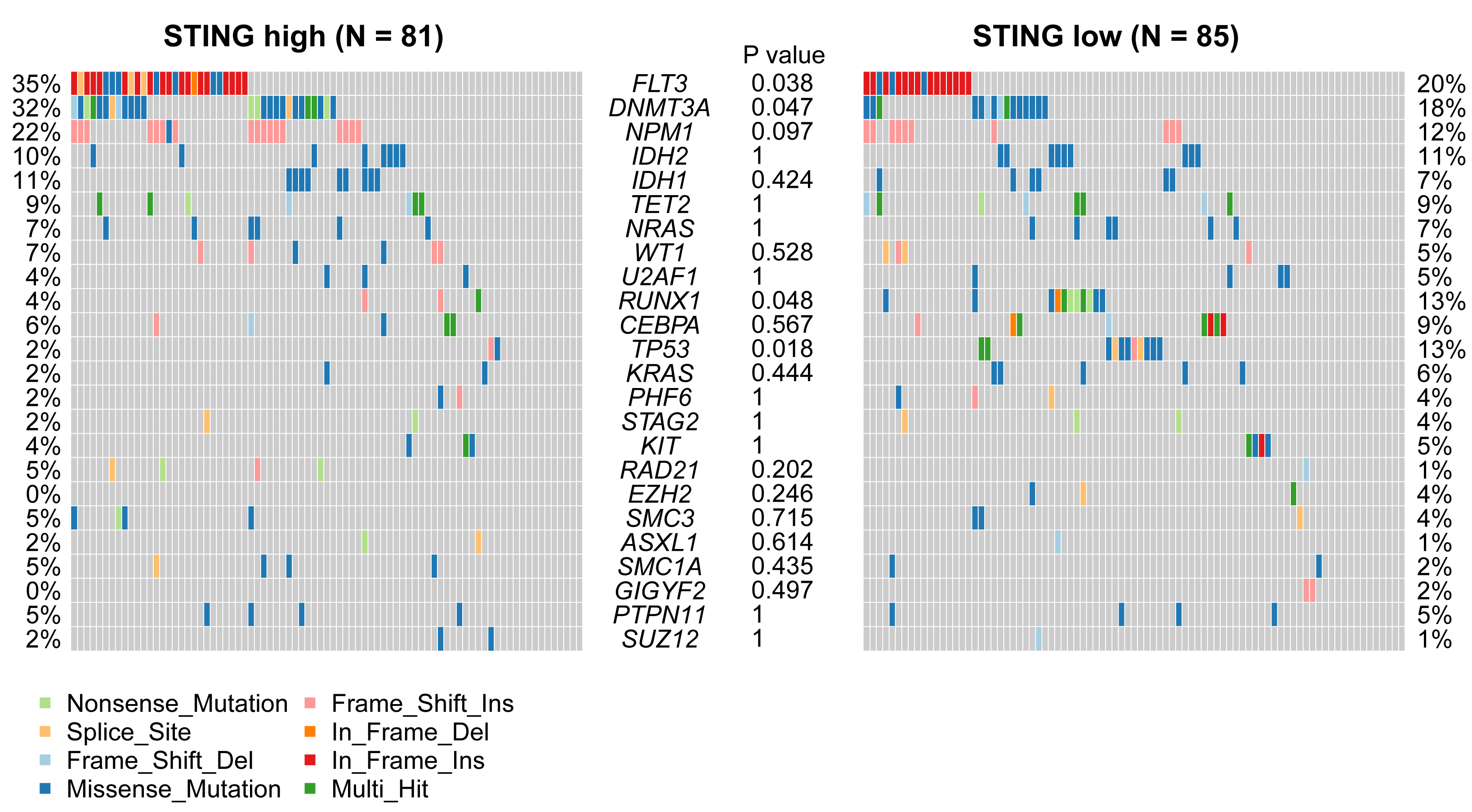

Supplement: Supplementary Figure 1 — Differences in somatic mutations between STING high and low groups in AML. The waterfall plot of somatic mutation features in patients with the high STING expression (A) or low STING expression (B) in the TCGA AML dataset. The mutation profiles showed the mutation difference of the 24 genes in high and low STING expression groups. [file Image1.tif]
